# *N*′-[(*E*)-3-Chloro-2-fluoro­benzyl­idene]-6-methyl­nicotinohydrazide monohydrate

**DOI:** 10.1107/S1600536812026736

**Published:** 2012-06-16

**Authors:** Hoong-Kun Fun, Ching Kheng Quah, P. C. Shyma, Balakrishna Kalluraya, J. H. S. Vidyashree

**Affiliations:** a X-ray Crystallography Unit, School of Physics, Universiti Sains Malaysia, 11800 USM, Penang, Malaysia; b Department of Studies in Chemistry, Mangalore University, Mangalagangotri, Mangalore 574 199, India

## Abstract

The title compound, C_14_H_11_ClFN_3_O·H_2_O, exists in an *E* conformation with respect to the N=C bond. The pyridine ring forms a dihedral angle of 5.00 (9)° with the benzene ring. In the crystal, the ketone O atom accepts one O—H⋯O and one C—H⋯O hydrogen bond, the water O atom accepts one N—H⋯O and two C—H⋯O hydrogen bonds and the pyridine N atom accepts one O—H⋯N hydrogen bond, forming layers parallel to the *ab* plane.

## Related literature

For general background to and the biological properties of hydrazone derivatives, see: Rollas & Kucukguzel (2007 ▶); Sondhi *et al.* (2009 ▶); Belskaya *et al.* (2010 ▶); Vijesh *et al.* (2011 ▶); Galil & Amr (2000 ▶). For standard bond-length data, see: Allen *et al.* (1987 ▶). For the stability of the temperature controller used in the data collection, see: Cosier & Glazer (1986 ▶). For related structures, see: Fun, Quah & Abdel-Aziz (2012 ▶); Fun, Quah, Shetty *et al.* (2012 ▶); Fun, Quah, Nitinchandra *et al.* (2012 ▶).
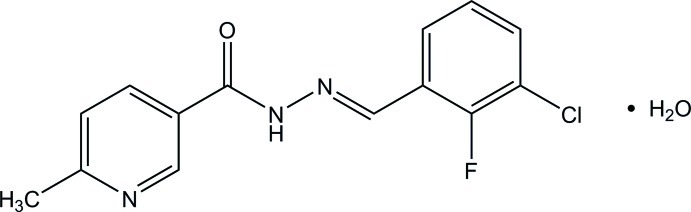


## Experimental

### 

#### Crystal data

C_14_H_11_ClFN_3_O·H_2_O
*M* *_r_* = 309.72
Monoclinic, 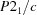

*a* = 9.7898 (12) Å
*b* = 6.4440 (8) Å
*c* = 23.121 (3) Å
β = 106.614 (5)°
*V* = 1397.7 (3) Å^3^
*Z* = 4
Mo *K*α radiation
μ = 0.29 mm^−1^
*T* = 100 K
0.47 × 0.24 × 0.13 mm

#### Data collection

Bruker SMART APEXII DUO CCD area-detector diffractometer
Absorption correction: multi-scan (*SADABS*; Bruker, 2009 ▶) *T*_min_ = 0.874, *T*_max_ = 0.962
12000 measured reflections
3154 independent reflections
2625 reflections with *I* > 2σ(*I*)
*R*_int_ = 0.031

#### Refinement

*R*[*F* ^2^ > 2σ(*F* ^2^)] = 0.046
*wR*(*F* ^2^) = 0.137
*S* = 1.05
3154 reflections
191 parameters
H-atom parameters constrained
Δρ_max_ = 0.43 e Å^−3^
Δρ_min_ = −0.63 e Å^−3^

### 

Data collection: *APEX2* (Bruker, 2009 ▶); cell refinement: *SAINT* (Bruker, 2009 ▶); data reduction: *SAINT*; program(s) used to solve structure: *SHELXTL* (Sheldrick, 2008 ▶); program(s) used to refine structure: *SHELXTL*; molecular graphics: *SHELXTL*; software used to prepare material for publication: *SHELXTL* and *PLATON* (Spek, 2009 ▶).

## Supplementary Material

Crystal structure: contains datablock(s) global, I. DOI: 10.1107/S1600536812026736/is5156sup1.cif

Structure factors: contains datablock(s) I. DOI: 10.1107/S1600536812026736/is5156Isup2.hkl

Supplementary material file. DOI: 10.1107/S1600536812026736/is5156Isup3.cml

Additional supplementary materials:  crystallographic information; 3D view; checkCIF report

## Figures and Tables

**Table 1:** Hydrogen-bond geometry (Å, °)

*D*—H⋯*A*	*D*—H	H⋯*A*	*D*⋯*A*	*D*—H⋯*A*
O1*W*—H2*W*1⋯O1^i^	0.78	2.11	2.8713 (18)	166
O1*W*—H1*W*1⋯N3^ii^	0.78	2.11	2.859 (2)	160
N2—H3⋯O1*W*	0.83	2.01	2.8104 (18)	162
C4—H4*A*⋯O1*W*	0.95	2.46	3.388 (2)	165
C7—H7*A*⋯O1*W*	0.95	2.39	3.1902 (19)	141
C12—H12*A*⋯O1^iii^	0.95	2.46	3.230 (2)	138

Symmetry codes: (i) 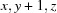; (ii) 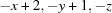; (iii) 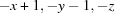.

**Fig. 1.**
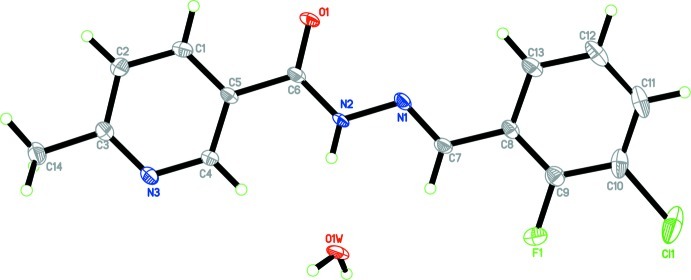
The molecular structure of the title compound showing 50% probability displacement ellipsoids for non-H atoms.

**Fig. 2.**
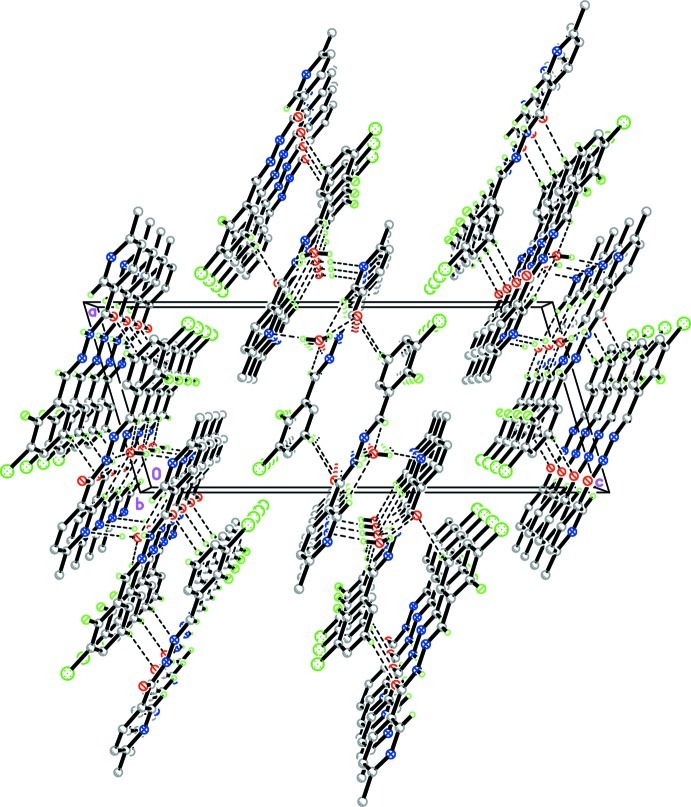
The crystal structure of the title compound, viewed along the b axis. H atoms not involved in hydrogen bonds (dashed lines) have been omitted for clarity.

## supplementary crystallographic information

### Comment

Hydrazones and their derivatives constitute a versatile class of compounds in organic chemistry. These compounds have showed varied biological properties, such as anti-inflammatory, analgesic, anticonvulsant, antituberculur, antitumor, anti-HIV and antimicrobial activity (Rollas & Kucukguzel, 2007; Sondhi *et al.*, 2009; Belskaya *et al.*., 2010). Hydrazones are important compounds for drug design, as possible ligands for metal complexes, and also for the syntheses of large number of heterocyclic compounds. Further, substituted pyridines have showed significant biological activities (Vijesh *et al.*, 2011; Galil & Amr, 2000). These reports prompted us to synthesize the novel derivative of 6-mthyl nicotinic acid hydrazide hydrazone to study its crystal structure.

The title compound (Fig. 1) consists of a *N*'-[(1*E*)-(3-chloro-2- fluorophenyl)methylidene]-6-methylnicotinohydrazide molecule and a water molecule in the asymmetric unit and exists in an *E* configuration with respect to the N1═C7 bond [1.279 (2) Å]. The pyridine ring (N3/C1–C5, r.m.s deviation = 0.008 Å) forms a dihedral angle of 5.00 (9)° with the benzene ring (C8–C13). Bond lengths (Allen *et al.*, 1987) and angles are within normal ranges and are comparable to related structures (Fun, Quah & Abdel-Aziz, 2012; Fun, Quah, Shetty *et al.*, 2012; Fun, Quah, Nitinchandra *et al.*, 2012).

In the crystal (Fig.2), molecules are linked *via* intermolecular O1W—H2W1···O1, C12—H12A···O1 bifurcated acceptor bonds (Table 1) and N2—H3···O1W, C4—H4A···O1W, C7—H7A···O1W trifurcated acceptor bonds and together with O1W—H1W1···N3 hydrogen bonds to form two-dimensional layers parallel to (001).

### Experimental

6-Methylnicotinohydrazide (1 g, 0.006 mol) and 3-chloro-2-flourobenzaldehyde (1.05 g, 0.006 mol) are refluxed for 1hr in ethanol(10 ml) by adding few drops of acetic acid. The solid separated on cooling was filtered, washed with chilled ethanol and dried. The crude material is recrystallized from hot ethanol(1.5 g, 78% ). *M.p.* : 457-458 °C. The crystals of appropriate size were obtained by the slow evaporation of the ethanolic solution of the compound.

### Refinement

N-bound and O-bound H atoms were located in a difference Fourier map and refined using a riding model with N—H = 0.8295 Å and O—H = 0.7778 or 0.7815 Å. The rest of hydrogen atoms were positioned geometrically and refined using a riding model with C—H = 0.95 or 0.98 Å and *U*_iso_(H) = 1.2 or 1.5 *U*_eq_(C). A rotating-group model was applied for the methyl group.

### Crystal data

**Table:**

C_14_H_11_ClFN_3_O·H_2_O	*F*(000) = 640
*M**_r_* = 309.72	*D*_x_ = 1.472 Mg m^−^^3^
Monoclinic, *P*2_1_/*c*	Mo *K*α radiation, λ = 0.71073 Å
Hall symbol: -P 2ybc	Cell parameters from 5275 reflections
*a* = 9.7898 (12) Å	θ = 3.2–30.0°
*b* = 6.4440 (8) Å	µ = 0.29 mm^−^^1^
*c* = 23.121 (3) Å	*T* = 100 K
β = 106.614 (5)°	Block, colourless
*V* = 1397.7 (3) Å^3^	0.47 × 0.24 × 0.13 mm
*Z* = 4	

### Data collection

**Table:**

Bruker SMART APEXII DUO CCD area-detector diffractometer	3154 independent reflections
Radiation source: fine-focus sealed tube	2625 reflections with *I* > 2σ(*I*)
Graphite monochromator	*R*_int_ = 0.031
φ and ω scans	θ_max_ = 27.5°, θ_min_ = 2.2°
Absorption correction: multi-scan (*SADABS*; Bruker, 2009)	*h* = −12→12
*T*_min_ = 0.874, *T*_max_ = 0.962	*k* = −8→8
12000 measured reflections	*l* = −30→30

### Refinement

**Table:**

Refinement on *F*^2^	Primary atom site location: structure-invariant direct methods
Least-squares matrix: full	Secondary atom site location: difference Fourier map
*R*[*F*^2^ > 2σ(*F*^2^)] = 0.046	Hydrogen site location: inferred from neighbouring sites
*wR*(*F*^2^) = 0.137	H-atom parameters constrained
*S* = 1.05	*w* = 1/[σ^2^(*F*_o_^2^) + (0.0777*P*)^2^ + 0.8539*P*] where *P* = (*F*_o_^2^ + 2*F*_c_^2^)/3
3154 reflections	(Δ/σ)_max_ = 0.001
191 parameters	Δρ_max_ = 0.43 e Å^−^^3^
0 restraints	Δρ_min_ = −0.63 e Å^−^^3^

### Special details

**Table:**

Experimental. The crystal was placed in the cold stream of an Oxford Cryosystems Cobra open-flow nitrogen cryostat (Cosier & Glazer, 1986) operating at 100.0 (1) K.
Geometry. All esds (except the esd in the dihedral angle between two l.s. planes) are estimated using the full covariance matrix. The cell esds are taken into account individually in the estimation of esds in distances, angles and torsion angles; correlations between esds in cell parameters are only used when they are defined by crystal symmetry. An approximate (isotropic) treatment of cell esds is used for estimating esds involving l.s. planes.
Refinement. Refinement of F^2^ against ALL reflections. The weighted R-factor wR and goodness of fit S are based on F^2^, conventional R-factors R are based on F, with F set to zero for negative F^2^. The threshold expression of F^2^ > 2sigma(F^2^) is used only for calculating R-factors(gt) etc. and is not relevant to the choice of reflections for refinement. R-factors based on F^2^ are statistically about twice as large as those based on F, and R- factors based on ALL data will be even larger.

### Fractional atomic coordinates and isotropic or equivalent isotropic displacement parameters (Å^2^)

**Table:**

	*x*	*y*	*z*	*U*_iso_*/*U*_eq_	
Cl1	0.13433 (5)	0.04401 (12)	−0.22498 (2)	0.0433 (2)	
F1	0.41013 (12)	0.18849 (16)	−0.14979 (5)	0.0253 (3)	
O1	0.90720 (13)	−0.28353 (18)	0.07189 (6)	0.0216 (3)	
N1	0.69521 (15)	−0.1028 (2)	−0.01138 (6)	0.0147 (3)	
N2	0.81273 (15)	0.0080 (2)	0.02094 (6)	0.0140 (3)	
H3	0.8209	0.1329	0.0139	0.017*	
N3	1.18040 (16)	0.3394 (2)	0.11873 (7)	0.0175 (3)	
C1	1.1389 (2)	−0.0710 (3)	0.14649 (8)	0.0213 (4)	
H1A	1.1260	−0.2118	0.1559	0.026*	
C2	1.2558 (2)	0.0394 (3)	0.17998 (8)	0.0224 (4)	
H2A	1.3235	−0.0246	0.2130	0.027*	
C3	1.27378 (18)	0.2453 (3)	0.16504 (8)	0.0158 (3)	
C4	1.06603 (18)	0.2327 (3)	0.08720 (8)	0.0164 (3)	
H4A	0.9987	0.3015	0.0551	0.020*	
C5	1.04020 (18)	0.0268 (2)	0.09874 (7)	0.0134 (3)	
C6	0.91479 (18)	−0.0965 (2)	0.06299 (7)	0.0144 (3)	
C7	0.60432 (18)	−0.0013 (2)	−0.05240 (7)	0.0145 (3)	
H7A	0.6211	0.1397	−0.0602	0.017*	
C8	0.47374 (17)	−0.1065 (3)	−0.08721 (7)	0.0146 (3)	
C9	0.37845 (19)	−0.0051 (3)	−0.13481 (8)	0.0182 (4)	
C10	0.25093 (19)	−0.0955 (3)	−0.16783 (8)	0.0243 (4)	
C11	0.2185 (2)	−0.2941 (3)	−0.15368 (9)	0.0276 (4)	
H11A	0.1321	−0.3580	−0.1760	0.033*	
C12	0.3126 (2)	−0.3998 (3)	−0.10675 (9)	0.0254 (4)	
H12A	0.2903	−0.5365	−0.0970	0.030*	
C13	0.43891 (19)	−0.3077 (3)	−0.07395 (8)	0.0191 (4)	
H13A	0.5027	−0.3824	−0.0420	0.023*	
C14	1.4001 (2)	0.3715 (3)	0.19936 (8)	0.0216 (4)	
H14A	1.3685	0.5111	0.2064	0.032*	
H14B	1.4689	0.3811	0.1759	0.032*	
H14C	1.4453	0.3046	0.2382	0.032*	
O1W	0.78116 (14)	0.41994 (18)	−0.02068 (6)	0.0207 (3)	
H2W1	0.8016	0.5100	0.0026	0.031*	
H1W1	0.7882	0.4580	−0.0518	0.031*	

### Atomic displacement parameters (Å^2^)

**Table:**

	*U*^11^	*U*^22^	*U*^33^	*U*^12^	*U*^13^	*U*^23^
Cl1	0.0185 (3)	0.0809 (5)	0.0264 (3)	0.0020 (3)	−0.00018 (19)	0.0134 (3)
F1	0.0256 (6)	0.0188 (5)	0.0300 (6)	0.0021 (4)	0.0052 (4)	0.0078 (4)
O1	0.0211 (7)	0.0072 (6)	0.0320 (7)	−0.0027 (5)	0.0004 (5)	0.0036 (5)
N1	0.0146 (7)	0.0096 (6)	0.0195 (7)	−0.0031 (5)	0.0040 (5)	−0.0031 (5)
N2	0.0149 (7)	0.0062 (6)	0.0194 (7)	−0.0033 (5)	0.0025 (5)	−0.0010 (5)
N3	0.0166 (7)	0.0121 (7)	0.0221 (7)	−0.0031 (5)	0.0027 (6)	−0.0012 (5)
C1	0.0214 (9)	0.0125 (8)	0.0270 (9)	−0.0021 (7)	0.0024 (7)	0.0045 (7)
C2	0.0175 (9)	0.0190 (9)	0.0257 (9)	−0.0014 (7)	−0.0018 (7)	0.0053 (7)
C3	0.0149 (8)	0.0142 (8)	0.0186 (8)	−0.0015 (6)	0.0053 (6)	−0.0020 (6)
C4	0.0162 (8)	0.0109 (7)	0.0198 (8)	−0.0018 (6)	0.0018 (6)	0.0015 (6)
C5	0.0135 (8)	0.0094 (7)	0.0181 (8)	−0.0016 (6)	0.0057 (6)	−0.0014 (6)
C6	0.0149 (8)	0.0091 (7)	0.0197 (8)	−0.0022 (6)	0.0058 (6)	−0.0006 (6)
C7	0.0164 (8)	0.0088 (7)	0.0182 (8)	−0.0014 (6)	0.0050 (6)	−0.0011 (6)
C8	0.0142 (8)	0.0122 (7)	0.0185 (7)	−0.0025 (6)	0.0065 (6)	−0.0040 (6)
C9	0.0177 (8)	0.0181 (8)	0.0199 (8)	−0.0011 (7)	0.0070 (6)	−0.0010 (6)
C10	0.0139 (8)	0.0409 (11)	0.0181 (8)	−0.0028 (8)	0.0045 (6)	−0.0038 (8)
C11	0.0173 (9)	0.0395 (12)	0.0276 (10)	−0.0127 (8)	0.0090 (7)	−0.0136 (8)
C12	0.0251 (10)	0.0204 (9)	0.0348 (10)	−0.0109 (8)	0.0153 (8)	−0.0099 (8)
C13	0.0204 (9)	0.0130 (8)	0.0251 (9)	−0.0035 (7)	0.0088 (7)	−0.0024 (6)
C14	0.0182 (9)	0.0209 (9)	0.0235 (8)	−0.0044 (7)	0.0023 (7)	−0.0033 (7)
O1W	0.0282 (7)	0.0081 (5)	0.0244 (6)	−0.0042 (5)	0.0052 (5)	0.0019 (5)

### Geometric parameters (Å, º)

**Table:**

Cl1—C10	1.731 (2)	C5—C6	1.497 (2)
F1—C9	1.354 (2)	C7—C8	1.467 (2)
O1—C6	1.228 (2)	C7—H7A	0.9500
N1—C7	1.279 (2)	C8—C9	1.386 (2)
N1—N2	1.3786 (18)	C8—C13	1.397 (2)
N2—C6	1.357 (2)	C9—C10	1.391 (2)
N2—H3	0.8295	C10—C11	1.380 (3)
N3—C3	1.338 (2)	C11—C12	1.385 (3)
N3—C4	1.338 (2)	C11—H11A	0.9500
C1—C2	1.381 (2)	C12—C13	1.386 (2)
C1—C5	1.393 (2)	C12—H12A	0.9500
C1—H1A	0.9500	C13—H13A	0.9500
C2—C3	1.395 (2)	C14—H14A	0.9800
C2—H2A	0.9500	C14—H14B	0.9800
C3—C14	1.503 (2)	C14—H14C	0.9800
C4—C5	1.391 (2)	O1W—H2W1	0.7778
C4—H4A	0.9500	O1W—H1W1	0.7815
			
C7—N1—N2	115.64 (14)	C9—C8—C13	117.44 (15)
C6—N2—N1	117.43 (13)	C9—C8—C7	120.04 (15)
C6—N2—H3	122.0	C13—C8—C7	122.51 (15)
N1—N2—H3	120.6	F1—C9—C8	119.08 (15)
C3—N3—C4	118.47 (15)	F1—C9—C10	118.73 (16)
C2—C1—C5	119.13 (16)	C8—C9—C10	122.19 (17)
C2—C1—H1A	120.4	C11—C10—C9	119.29 (18)
C5—C1—H1A	120.4	C11—C10—Cl1	121.11 (15)
C1—C2—C3	119.62 (16)	C9—C10—Cl1	119.59 (16)
C1—C2—H2A	120.2	C10—C11—C12	119.69 (17)
C3—C2—H2A	120.2	C10—C11—H11A	120.2
N3—C3—C2	121.56 (15)	C12—C11—H11A	120.2
N3—C3—C14	116.62 (15)	C11—C12—C13	120.50 (18)
C2—C3—C14	121.81 (16)	C11—C12—H12A	119.8
N3—C4—C5	123.74 (15)	C13—C12—H12A	119.8
N3—C4—H4A	118.1	C12—C13—C8	120.87 (17)
C5—C4—H4A	118.1	C12—C13—H13A	119.6
C4—C5—C1	117.45 (15)	C8—C13—H13A	119.6
C4—C5—C6	124.46 (15)	C3—C14—H14A	109.5
C1—C5—C6	118.08 (15)	C3—C14—H14B	109.5
O1—C6—N2	122.65 (15)	H14A—C14—H14B	109.5
O1—C6—C5	120.50 (15)	C3—C14—H14C	109.5
N2—C6—C5	116.85 (14)	H14A—C14—H14C	109.5
N1—C7—C8	118.86 (15)	H14B—C14—H14C	109.5
N1—C7—H7A	120.6	H2W1—O1W—H1W1	109.3
C8—C7—H7A	120.6		
			
C7—N1—N2—C6	−177.06 (15)	N2—N1—C7—C8	−177.73 (13)
C5—C1—C2—C3	−0.7 (3)	N1—C7—C8—C9	−175.30 (16)
C4—N3—C3—C2	1.4 (3)	N1—C7—C8—C13	5.6 (3)
C4—N3—C3—C14	−179.66 (15)	C13—C8—C9—F1	−178.62 (15)
C1—C2—C3—N3	−0.1 (3)	C7—C8—C9—F1	2.2 (2)
C1—C2—C3—C14	−179.06 (18)	C13—C8—C9—C10	1.4 (3)
C3—N3—C4—C5	−1.8 (3)	C7—C8—C9—C10	−177.74 (16)
N3—C4—C5—C1	0.9 (3)	F1—C9—C10—C11	178.90 (16)
N3—C4—C5—C6	−178.41 (16)	C8—C9—C10—C11	−1.1 (3)
C2—C1—C5—C4	0.4 (3)	F1—C9—C10—Cl1	−2.2 (2)
C2—C1—C5—C6	179.74 (16)	C8—C9—C10—Cl1	177.77 (14)
N1—N2—C6—O1	1.7 (3)	C9—C10—C11—C12	0.4 (3)
N1—N2—C6—C5	−178.62 (13)	Cl1—C10—C11—C12	−178.47 (15)
C4—C5—C6—O1	172.25 (17)	C10—C11—C12—C13	0.0 (3)
C1—C5—C6—O1	−7.1 (3)	C11—C12—C13—C8	0.4 (3)
C4—C5—C6—N2	−7.4 (2)	C9—C8—C13—C12	−1.0 (3)
C1—C5—C6—N2	173.25 (15)	C7—C8—C13—C12	178.11 (16)

### Hydrogen-bond geometry (Å, º)

**Table:**

*D*—H···*A*	*D*—H	H···*A*	*D*···*A*	*D*—H···*A*
O1*W*—H2*W*1···O1^i^	0.78	2.11	2.8713 (18)	166
O1*W*—H1*W*1···N3^ii^	0.78	2.11	2.859 (2)	160
N2—H3···O1*W*	0.83	2.01	2.8104 (18)	162
C4—H4*A*···O1*W*	0.95	2.46	3.388 (2)	165
C7—H7*A*···O1*W*	0.95	2.39	3.1902 (19)	141
C12—H12*A*···O1^iii^	0.95	2.46	3.230 (2)	138

Symmetry codes: (i) *x*, *y*+1, *z*; (ii) −*x*+2, −*y*+1, −*z*; (iii) −*x*+1, −*y*−1, −*z*.
